# Community engagement to improve OHCA outcomes: The “Heart Safe Beach” initiative – Timmendorfer Strand Germany

**DOI:** 10.1016/j.resplu.2025.100979

**Published:** 2025-05-12

**Authors:** Benito Baldauf, Justin Große Feldhaus, Jana Hummel, Hendrik Bonnemeier

**Affiliations:** Christian-Albrechts University Kiel, Germany; Institute of Life Sciences, Hochschule Bremerhaven, Germany; Klinikum Ostfriesland, Germany; Institute of Life Sciences, Hochschule Bremerhaven, Germany; Christian-Albrechts University Kiel, Germany; Institute of Life Sciences, Hochschule Bremerhaven, Germany; University Hospital Rostock, Germany

To the Editor,

Sudden cardiac death (SCD) remains a leading cause of mortality worldwide.^1^ Survival hinges on immediate resuscitation efforts and rapid defibrillation, underscoring the importance of layperson intervention.^2^ To address these critical needs, the *Heart Safe Beach* initiative was launched, aiming to (1) enhance lay resuscitation efforts, (2) increase automated external defibrillator (AED) availability, and (3) improve emergency dispatch efficiency.

**Heart Safe Beach Initiative Overview**

**Table:** 

Component	Action Taken	Early Results
**Public Training**	Layperson CPR and AED courses, on- and off-season sessions	>400 individuals trained
**AED Deployment**	22 AEDs installed based on OHCA heat map analysis (Figure S1)	100–150 m maximum distance between AEDs
**Awareness Campaign**	Posters in public spaces; local media engagement	Increased community recognition reported
**EMS Integration**	AED sites integrated into EMS dispatch system with GPS tracking	Expected faster response; ongoing evaluation

To evaluate Aim 1, training records document the number of laypersons trained. Public awareness was promoted through campaigns featuring posters, flyers, and media coverage as planned in the implementation algorithm (Fig. 1). The initiative aligns AED placement with OHCA incidence heat maps, aiming for <3-minute access on foot.

**Fig. 1:**
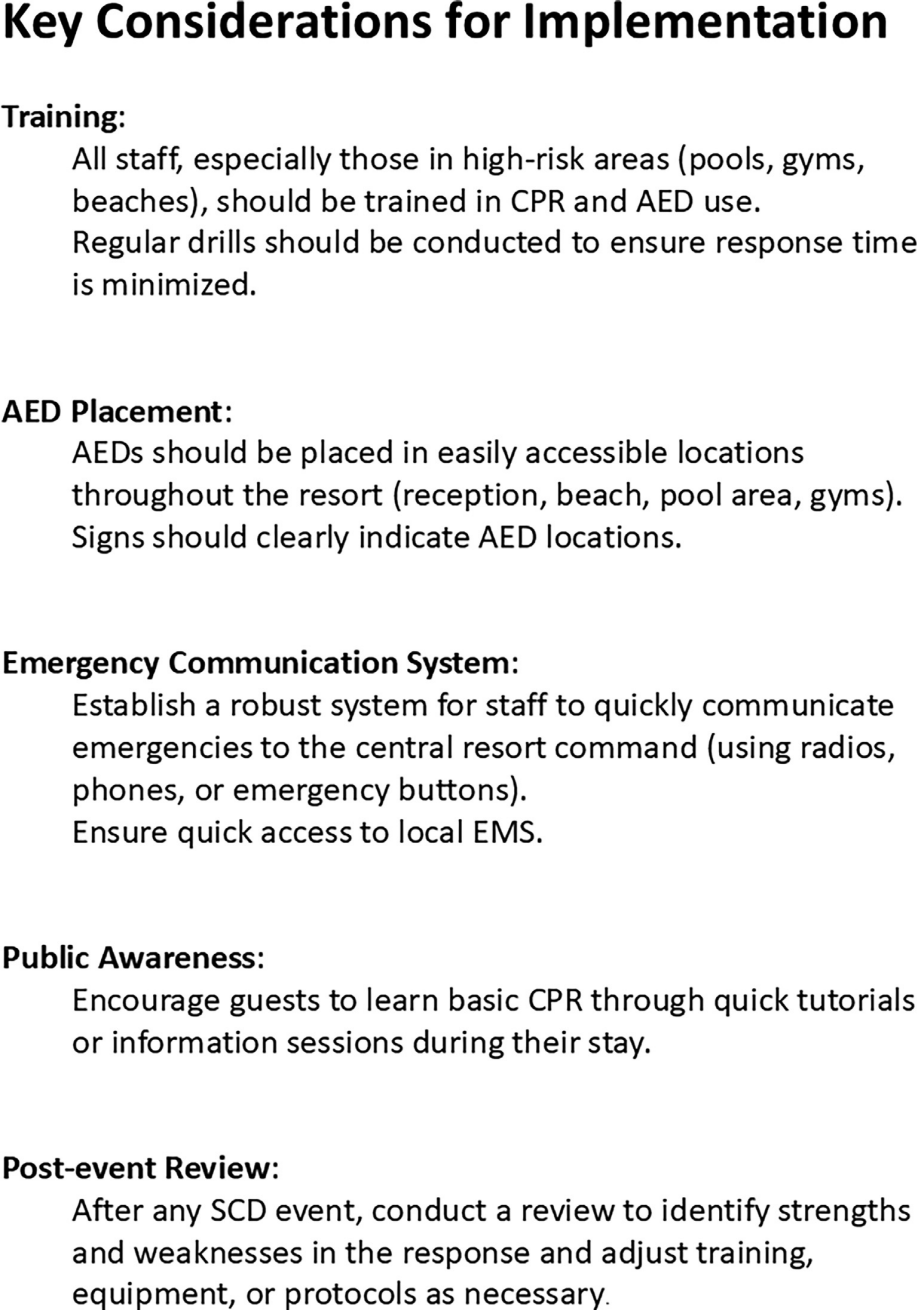
*Implementation algorithm of the “Heart Safe Beach” initiative in Timmendorfer Strand*. This algorithm outlines the stepwise development and execution of the community-based intervention aimed at improving out-of-hospital cardiac arrest (OHCA) outcomes. The algorithm highlights key stages including stakeholder engagement, training dissemination, AED infrastructure planning, public awareness strategies, and ongoing evaluation. This overview provides a structured framework to guide similar beachside or public area initiatives globally.

In the first phase, 122 OHCA cases were identified and mapped (Figure S1). Strategic AED placement based on these data improved coverage across high-risk areas. Early feedback from first responders and community surveys indicates an improvement in lay preparedness, though a formal outcome evaluation (e.g., bystander CPR rates) is ongoing.

We acknowledge the need for more robust outcome metrics, such as documented increases in bystander CPR or AED use, and are actively working on integrating these data into registry reporting systems for future evaluation phases.

We hope this initiative encourages similar community-based models for improving survival from OHCA.

## Ethics approval and consent to participate

Not applicable.

## Consent for publication

Not applicable.

## Availability of data and materials

All data generated or analyzed during this study are included in this published article.

## Clinical trial number

Not applicable.

## CRediT authorship contribution statement

**Benito Baldauf:** Writing – original draft, Validation, Supervision, Data curation. **Justin Große Feldhaus:** Writing – review & editing, Project administration, Methodology, Investigation, Formal analysis, Data curation, Conceptualization. **Jana Hummel:** Writing – review & editing, Supervision, Funding acquisition. **Hendrik Bonnemeier:** Writing – original draft, Validation, Supervision, Resources, Project administration, Methodology, Investigation, Funding acquisition, Formal analysis, Data curation, Conceptualization.

## Funding

Supported by the DEAL consortium agreement between the German Rectors Conference and Elsevier.

## Declaration of competing interest

The authors declare that they have no known competing financial interests or personal relationships that could have appeared to influence the work reported in this paper.
