# Supplementary figures and images for: Community engagement to improve OHCA outcomes: The “Heart Safe Beach” initiative – Timmendorfer Strand Germany
Source: Resusc Plus. 2025 May 12;24:100979. doi: 10.1016/j.resplu.2025.100979 (PMC12149431; doi:10.1016/j.resplu.2025.100979)

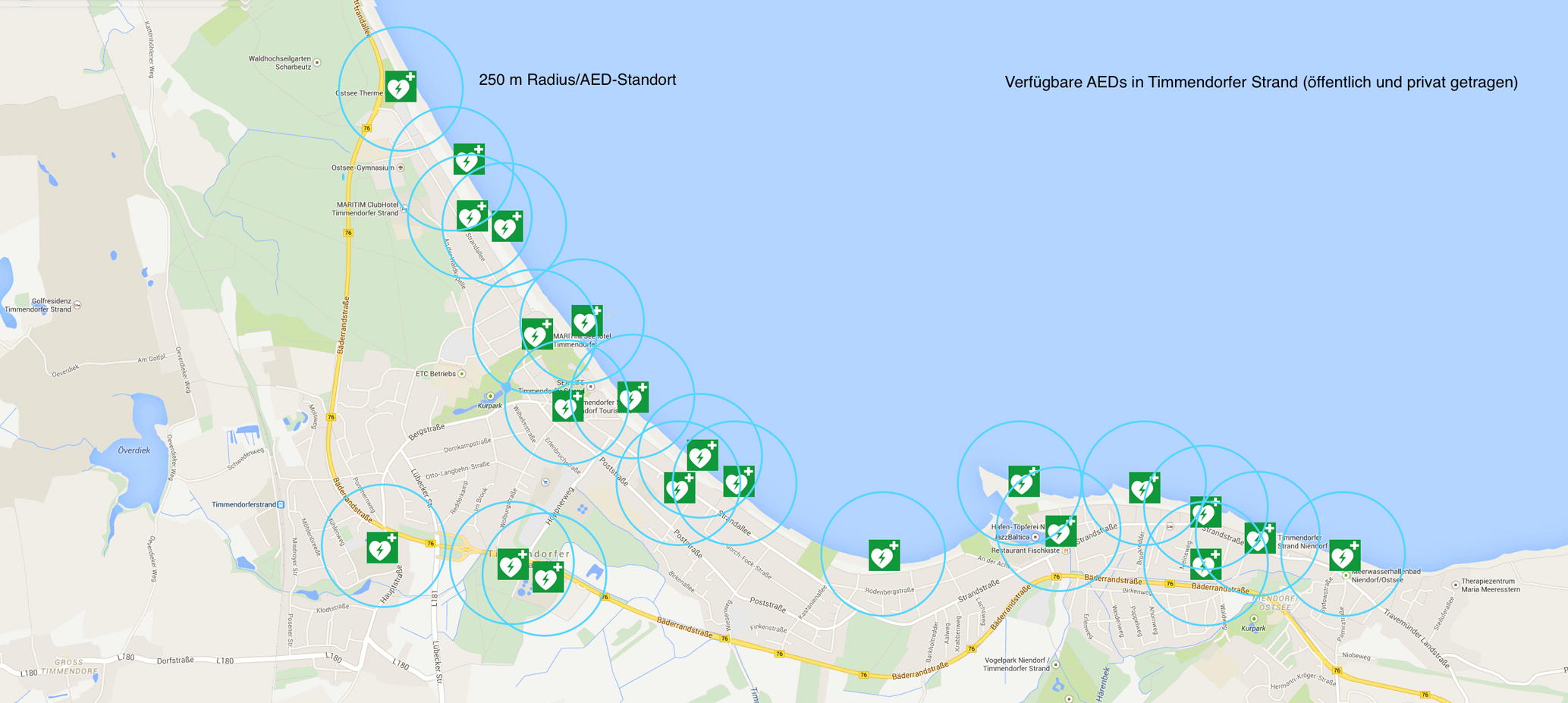

Supplement: Supplementary Data 1 [file mmc1.jpg]
